# Supplementary material for: Genetic Mechanism for Antioxidant Activity of Endogenous Enzymes under Salinity and Temperature Stress in Turbot (Scophthalmus maximus)
Source: Antioxidants (Basel). 2022 Oct 19;11(10):2062. doi: 10.3390/antiox11102062 (PMC9598745; doi:10.3390/antiox11102062)
Supplement: Supplementary file 1 [file antioxidants-11-02062-s001.zip › antioxidants-1946190-supplementary.pdf]

Table S1 Activities of CAT, SOD and GPX of juvenile turbot *Scophthalmus maximus* in different salinity

**Salinity**	**Antioxidant enzymes /U/mg**	**Repeat group 1**	**Repeat group 2**	**Repeat group 3**
5‰	SOD	441.76	460.42	464.21
	CAT	1.62	1.74	1.89
	GPX	28.76	36.66	39.7
10‰	SOD	314.14	327.25	368.35
	CAT	1.43	1.51	1.53
	GPX	26.79	31.56	32.04
20‰	SOD	341.76	372.12	405.27
	CAT	1.57	1.57	1.63
	GPX	31.16	41.51	35.77
30‰	SOD	229.66	204.46	265.18
	CAT	1.11	0.87	1.02
	GPX	28.02	22.14	27.3
40‰	SOD	445.23	478.12	378.74
	CAT	2.27	2.13	1.93
	GPX	37.08	37.74	42.96

Table S2 Activities of CAT, SOD, and GPX in liver of juvenile turbot in different temperature

Temperature	Antioxidant enzymes/U/mg	Repeat group 1	Repeat group 2	Repeat group 3
17ºC	SOD	238.98	260.19	199.99
	CAT	1.04	0.85	1.11
	GPX	22.64	29.06	25.76
	SOD	116.53	99.86	108.45

20ºC	CAT	1.34	1.15	1.05
	GPX	56.11	78.86	48.54
	SOD	123.56	116.84	121.31
23ºC	CAT	1.55	1.44	1.87
	GPX	48.16	41.26	44.65
	SOD	155.94	151.43	164.29
25ºC	CAT	1.59	2.2	2.06
	GPX	80.44	88.46	80.19
	SOD	166.01	192.83	179.42
28ºC	CAT	1.09	1.04	0.93
	GPX	77.68	86.35	93.49
